# Sudden Sensorineural Hearing Loss and Polymorphisms in Iron Homeostasis Genes: New Insights from a Case-Control Study

**DOI:** 10.1155/2015/834736

**Published:** 2015-02-18

**Authors:** Alessandro Castiglione, Andrea Ciorba, Claudia Aimoni, Elisa Orioli, Giulia Zeri, Marco Vigliano, Donato Gemmati

**Affiliations:** ^1^Department of Neurosciences-Complex Operative Unit of Otorhinolaryngology and Otosurgery, University Hospital of Padua, Via Giustiniani 2, 35128 Padua, Italy; ^2^ENT & Audiology Department, University Hospital of Ferrara, Via Aldo Moro 8, 44124 Cona, Ferrara, Italy; ^3^Centre for Haemostasis & Thrombosis, Haematology Section, Department of Medical Sciences, University of Ferrara, 44100 Ferrara, Italy

## Abstract

*Background*. Even if various pathophysiological events have been proposed as explanations, the putative cause of sudden hearing loss remains unclear. *Objectives*. To investigate and to reveal associations (if any) between the main iron-related gene variants and idiopathic sudden sensorineural hearing loss. *Study Design*. Case-control study. *Materials and Methods*. A total of 200 sudden sensorineural hearing loss patients (median age 63.65 years; range 10–92) were compared with 400 healthy control subjects. The following genetic variants were investigated: the polymorphism c.−8CG in the promoter of the ferroportin gene (FPN1; *SLC40A1*), the two isoforms C1 and C2 (p.P570S) of the transferrin protein (*TF*), the amino acidic substitutions p.H63D and p.C282Y in the hereditary hemochromatosis protein (*HFE*), and the polymorphism c.–582AG in the promoter of the *HEPC* gene, which encodes the protein hepcidin (HAMP). *Results*. The homozygous genotype c.−8GG of the *SLC40A1* gene revealed an OR for ISSNHL risk of 4.27 (CI 95%, 2.65–6.89; *P* = 0.001), being overrepresented among cases. *Conclusions*. Our study indicates that the homozygous genotype *FPN1* −8GG was significantly associated with increased risk of developing sudden hearing loss. These findings suggest new research should be conducted in the field of iron homeostasis in the inner ear.

## 1. Introduction

Sudden sensorineural hearing loss (SSNHL) represents an acute inner ear disorder, mostly unilateral, that generally affects adults worldwide, even if higher occurrence rates are reported in developed countries [1, 2]. It has been estimated that SSNHL has an overall incidence rate of 5–20/100,000 individuals per year, though this is most likely an underestimate [2, 3]. Several pathophysiological mechanisms for idiopathic sudden sensorineural hearing loss (ISSNHL) have been proposed in the literature: local hypoxic/ischemic events (including coagulopathies, hypotension, and thromboembolism), autoimmune and metabolic disorders, inner ear viral infections, rupture of the inner ear membranes, free radicals induced-damage, neuronal damage, and dysregulation of the local inflammatory response [3–5] leading to transient or permanent dysfunction of cochlear microcirculation [6]. More than isolated events, they could be considered as part of a unique vicious circle determining a sudden clinical manifestation when local recovery or residual functions reach a subjective threshold or trigger point.

To contribute to the comprehension of the homeostatic events, the present study focused on divalent metallic ions and in particular on iron metabolism [7]. There is a growing interest in the association between iron metabolism and oxidative stress; as it has been reported iron excess is a potential cause of increased oxidative stress and therefore involved in cellular injury and death. Iron is an essential nutrient, but its divalent form (Fe^2+^), as well as other divalent metal ions, also has the capacity to enhance redox cycling and free radical formation [8, 9]. Iron-mediated oxidative stress is hypothesized to be involved in the pathogenesis of several degenerative disorders, including thrombosis, venous ulcers, chronic venous disease, and central nervous system disorders such as multiple sclerosis or neoplasms [10–13]. In addition, a novel iron-driven etiopathogenetic mechanism, responsible for increase in free radical generation, has been formulated in degenerative skin lesions appearance comprehending coexistence of local iron overload and iron homeostasis gene-variants [14]. Accordingly, similar conditions could be potentially associated with sudden hearing loss [15]. Therefore, divalent ions homeostasis could be a central pathway involved in the pathophysiology of sudden hearing loss [16, 17]. In fact, the one of the known models of hearing loss in Pendred syndrome is iron/free radical stress-mediated [18]. The main genes and their variants we selected and investigated with possible effects on tissue injury [10, 19] are described below.

The* HFE* gene (6p21.3–22.2) encodes a membrane protein of 348 amino acids that belongs to the MHC class I family. The protein is normally expressed in cryptal enterocytes of the duodenum, liver, placenta, kidney, plasma, and platelets. Complexing with beta 2 microglobulin and transferrin receptor (TFR2), it plays a crucial role in iron homeostasis, and, when mutated, it is believed to be responsible for hemochromatosis, variegate porphyria, and microvascular complications of diabetes. Variants and/or mutated proteins bind to the transferrin receptor and reduce its affinity for iron-loaded transferrin. Thus, they finally unbalance the iron intake. Recent increasing interest in neurodegenerative disorders mediated by iron overload has highlighted the* HFE* gene among candidates for further investigations into iron homeostasis involvement in similar conditions. A review of the literature suggests a potential role even in iron-mediated hearing loss also in the past [20, 21]. Finally, it is important to note that the* HFE* gene is also expressed in the brain, spinal cord, cortex, and cerebellum [22, 23]. Thus, it could be directly involved or responsible for specific conditions concerning the central nervous system.

The* FPN1* (*SLC40A1*) gene is located on chromosome 2 (2q32), and it encodes a protein (ferroportin) of 570 amino acids with the specific function of exporting iron out from cells in the basolateral space, except for the reticuloendothelial cells, which can spread iron ions into the blood circulation. Consequently,* FPN1* plays a crucial role in iron homeostasis [24], and, generally, it has the opposite function of the* DMT1* protein product [25], which allows intracellular passage of divalent iron (Fe^2+^). Ferroportin is expressed in different tissues, though to the best of our knowledge there are no previous reported studies that document its localization into the cochlea. Similar to other iron genes, it undergoes iron regulation at the transcriptional and translational level [26–28].

Another important gene related to ferroportin expression and function is the* HEPC *(19q13.1) gene encoding the protein hepcidin (HAMP). This protein is a 25-amino-acid peptide, derived from the cleavage of an 84-amino-acid long propeptide that is mainly synthesized by hepatocytes and is a major regulator of iron balance activity via binding to the FPN1 protein on the cell membrane, suppressing it [29, 30].* HEPC* expression and its role in iron homeostasis may play a crucial role in different conditions [31].

The* TF* gene (3q22.1) encodes for the protein transferrin, which forms a stable complex with the HFE protein, which facilitates iron transfer via the transferrin receptor. The function of this protein is to transport iron from the intestine, reticuloendothelial system, and liver parenchymal cells to all proliferating cells in the body. The effect of HFE on iron absorption depends on its relationship with the transferrin receptor. HFE variants affect TF binding, determining a loss of HFE-repressor function for TF uptake, thereby increasing iron transport within the cells.

As there are no previous reports in the literature about the role of iron metabolism regulatory genes in the pathophysiology of SSNHL, we proceeded to evaluate if specific single nucleotide polymorphisms within the main iron genes are potentially involved in the susceptibility to SSNHL.

## 2. Materials and Methods

### 2.1. Study Design Patient and Control Enrolment

The research was designed as a case-control study. A total of 200 patients (104 females and 96 males), affected by idiopathic sudden sensorineural hearing loss (ISSNHL) and referred to the ENT and Audiology Department of the University Hospital of Ferrara from 2009 to 2013, were enrolled for this study. According to the current literature, ISSNHL was defined as a sudden hearing loss (≥30 dB HL), within 3 consecutive frequencies, developing over 72 hours [32].

All patients underwent a complete clinical interview with a complete audiological evaluation, including microotoscopy, tonal and speech audiometry, impedancemetry, auditory brainstem responses (ABRs), and MRI with gadolinium to rule out retrocochlear pathology.

The patients were enrolled in the study after diagnosis of ISSNHL made by experienced audiologists. Exclusion criteria were specific causes of sudden hearing impairment such as meningitis, traumas, or surgery outcomes and complications.

For each patient, a Pure Tone Average hearing threshold (PTA dBHL) was estimated from the hearing levels at 0.5, 1, 2, and 4 kHz. The average degree of hearing loss was then classified into the following groups, according to a modified method from Clark [33]: normal hearing (10–15 dB HL), mild hearing loss (16–40 dB HL), moderate hearing loss (41–70 dB HL), severe hearing loss (71–90 dB HL) and profound hearing loss (>90 dB HL). The type of hearing loss was then evaluated according to the Tran Ba Huy classification [34] and thereby stratified into five types: A, B, C, D, and E.

The control group consisted of 400 healthy volunteers with no personal or familial history of previous SSNHL and they were completely matched with the case group by sex, age, and ethnicity.

### 2.2. Audiological Equipment

Pure tone threshold audiometry was performed within a sound-proof cabin (model E2X2, roll 01008 220V 10A; Mercury, Milan, Italy) using an Amplaid audiometer (Amplaid, Milan, Italy), calibrated to ISO 9001 standards. The audiometric procedure was performed using headphones to assess air conduction and a bone vibrator for bone conduction. The better ear was evaluated first. An ascending method using 5 dB HL steps was utilized to calculate hearing threshold. Air conduction hearing thresholds were assessed at 125, 250, 500, 1000, 2000, 4000, and 8000 Hz. Bone conduction hearing thresholds were assessed with the use of a masking, white, and contralateral noise, at 250, 500, 1000, 2000, and 4000 Hz.

### 2.3. Genetic Investigations

All enrolled patients (*n* = 200) underwent DNA analysis for the identification of specific genetic variants in 4 iron-related genes:* FPN1* (*SLC40A1*) (ferroportin),* TF* (transferrin),* HFE* (human hemochromatosis gene), and* HEPC/HAMP* (hepcidin). All these genes are known to play a crucial role in iron homeostasis with increasing evidence of involvement in inner ear pathophysiology.

The following polymorphisms were examined:

- *FPN1*, 5′UTR c.−8CG,
- *TF*, C1 and C2 variants (p.P570S),
- *HFE* p.H63D and p.C282Y,
- *HEPC, *5′UTR c.−582AG.

DNA was isolated from peripheral frozen whole blood by an automated DNA extraction and purification robot (BioRobot EZ1 system from QIAGEN; Hilden, Germany), which performs purification of nucleic acids using a magnetic bead technology.

PCR amplifications were performed by means of the lyophilic complete UNIVERSAL MASTER MIX kit (STAT-NAT DNA-Mix; SENTINEL Diagnostics, Milan, Italy) containing all the reagents useful to amplify the extracted DNA with the exclusion of the specific pair of primers. The PCR cycle for the different SNP containing fragments was as follows:* HFE, FPN1, HEPC*, and* TF* (one cycle of denaturing at 95°C/5 min followed by 33 cycles of 94°/30 sec; 57°/30 sec; 72°/60 sec). PCRs were performed in a PTC-200 thermal cycler (M. J. Research, Inc., Watertown, MA, USA). SNP detection was performed using the Pyromark ID System (Biotage AB Uppsala, Sweden) according to the standard procedures for amplicon denaturation, purification, and sequencing following the manufacturer's recommendations. All the oligo sequences of the SNPs investigated (Forward, Reverse, and Sequence primers) were selected to have an at least 98.0% compatibility score.

Haplotypes were confirmed by regenotyping of approximately 20% of randomly selected samples among each different genotype group for each specific polymorphism by means of enzymatic restriction of PCR amplicons. All the digestion reactions were performed according to the supplier's instructions. There were no discrepancies between genotypes determined in duplicate and/or by different methods. Known genotypes were used as control references. Additional nonreported technical details are as previously described [10].

Written informed consent was obtained from all participants according to current national rules and laws for publication. All further research was conducted in accordance with the ethical standards of all applicable national and institutional committees and with the World Medical Association's Helsinki Declaration.

### 2.4. Statistical Analysis

Significant differences among groups were assessed by chi-square test for genotype distribution comparisons. When appropriate, Yates' correction or Fisher's exact test was applied. Adjusted odds ratios (OR) and 95% confidence intervals (95% CI), calculated by logistic regression models were used to estimate the risk associated with the presence of the rare homozygous condition (e.g.,* FPN1 −8GG*,* Tf C2C2*,* HFE 63DD*, and* HEPC-582GG*) or with the heterozygotes (i.e.,* HFE 282CY*) compared with the remaining genotypes (i.e., heterozygous and/or homozygous for the common allele) in cases versus the controls according to a recessive model of phenotype expression.

## 3. Results

### 3.1. Single Analyses

A total of 200 patients affected by ISSNHL and referred to the ENT and Audiology Department of the University Hospital of Ferrara between January 2009 and December 2013 were included in this study. Of these, 104 (52%) were females and 96 (48%) were males; quietly all (*n* = 198; 99%) were unilateral cases (103; 51.5% right side and 95; 47.5% left side), and 2 (1%) cases were bilateral; the median age was 63.65 years (age range: 10–92 years) (Table 1).

The DNA of the 200 patients was sampled and analyzed to identify the polymorphism −8CG of the* FPN1* gene promoter region. A total of 123 (61.5%) patients were −8CC homozygotes, 62 (31%) were heterozygotes, and 15 (7.5%) were −8GG homozygotes.

The same 200 patients were searched for the variant H63D of the* HFE* gene: 56 (28%) were heterozygous; 2 (1%) cases were 63DD homozygotes, and the remaining 142 (71%) were 63HH homozygotes.

Concerning the polymorphism analysis for the C282Y* HFE* gene, we identified 7 (3.5%) cases as 282CY heterozygotes and no 282YY homozygous were found. Furthermore, 12 (6%) cases presented the polymorphism −582AG in the* HEPC* gene in homozygosis (GG) and 77 (38.5%) in heterozygosis (AG), whereas the remaining 111 (55.5%) cases did not present the genetic variant.

Finally, the presence of the two variants C1 and C2 of the transferrin protein yielded 8 (4%) patients C2C2 homozygotes, 53 (26.5%) C1C2 heterozygotes, and 139 (69.5%) C1C1 homozygotes for the isoforms (Table 2).

The homozygous genotype c.−8GG of the* SLC40A1* gene revealed an OR for ISSNHL risk of 4.27 (CI 95%, 2.65–6.89; *P* = 0.001), being significantly overrepresented among cases. No significant ORs were observed under the additive or dominant/recessive models for the analysis in the other genes investigated.

### 3.2. Combined Analyses

In attempt to calculate a cumulative HL risk associated with the coexistence of multiple predisposing genotypes, we compared the whole group of cases and controls carrying a combination of different polymorphic alleles. In detail, subjects carrying at least four “risk-alleles” in at least two different SNPs (multicarriers) were computed and compared with subjects who were homozygous for the common allele in all the considered gene variants (fully wild-types). Combined homozygotes at least in two different SNPs, single homozygotes in one, and combined carriers in at least two, or carrying at least a quadruple heterozygous condition, they globally were 7.0% in patients (*n* = 14) and 4.75% in controls (*n* = 19). Conversely, the fully wild-type condition was 18.5% in cases (*n* = 37) and 19.0% in controls (*n* = 76). Although the slight trend observed in the overrepresentation of the rare at risk combined alleles, no significant risk-values were obtained from this kind of comparison (OR = 1.51; CI 95%, 0.68–3.35) Finally, stratifying the groups of patients by their hearing loss score, no significant correlation was observed between a particular allele and the clinical severity (Table 3).

## 4. Discussion

**Figure 1:**
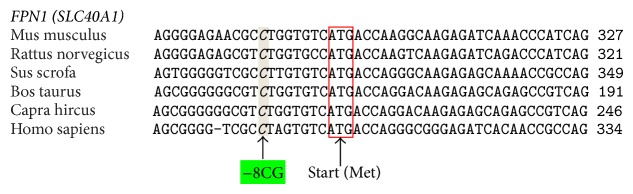
The panel shows how much the promoter region of* SLC40A1* gene containing the polymorphic nucleotide is conserved among different kinds of mammals. ClustalW2 http://www.ebi.ac.uk/.

**Figure 2:**
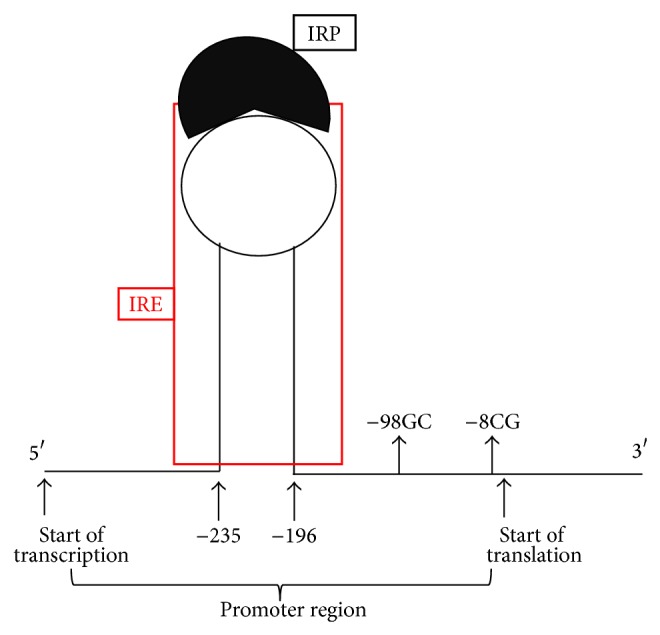
Schematic representation of the hairpin of the iron regulatory element (IRE) interacting with the iron regulatory protein (IRP) in the* FPN1* promoter region. It is to note the closeness of the −98GC SNP to the “beginning” of the IRE sequence.

**Figure 3:**
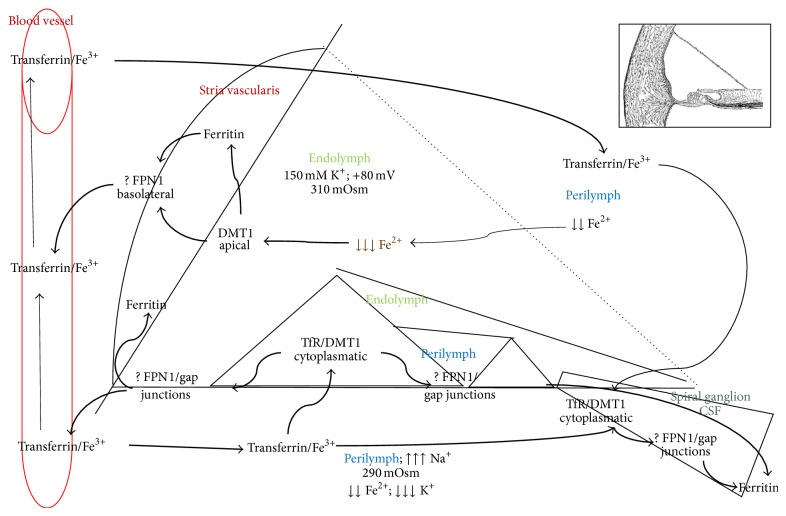
Divalent metal ion homeostasis in the inner ear. The picture shows a schematic representation of a section of the inner ear; reviewing the literature allowed the speculation around the potential involvement of specific proteins in the iron homeostasis. Considering distribution and function of those proteins it may be possible that iron ions follow a cycle similar to the potassium cycle, also through gap junctions. In particular, transferrin is concentrated in perilymph and cerebrospinal fluid and it transports trivalent (ferric) ions from and to blood vessels. In addition iron ions are stocked in the stria vascularis (ferritin depots). In fact, the apical expression of DMT1 is reasonably involved in reabsorption of free divalent ions from inner ear fluids thus maintaining electrochemical features. Cytoplasmic DMT1 allows the uptake of molecules of transferrin through Tf receptors. Ferroportin (FPN 1) is basolaterally located and probably helps inner ear cells in the reuptake of divalent ions, taking them away from the sensorineural epithelium during acoustic stimulation to avoid free radical damage. Ions are taken off through the basolateral part of cells and “pushed” towards spiral ganglion and stria vascularis. It is possible that divalent ions pass through gap junctions (metal divalent ion diameter could be less than 1 nanometer; thus molecules can passively diffuse through connexons in the intercellular space). Summarizing, divalent ions are transported from area with few blood vessels and low ferritin concentrations, to the structures that can storage or evacuate divalent ions, such as stria vascularis. Outer and inner ear cells may introduce a small amount of divalent ions through calcium channels.

Sudden hearing loss has been reported to be associated with a huge number of clinical conditions [35–38]. The uncertainty of reasonable etiologies has encouraged continuous investigations aimed at identifying the most convincing pathological explanation. Moreover, it has encouraged new considerations of the predisposing genetic conditions that might explain the clinical variability when a positive correlation with factorial events occurs. Far from the identification of an unequivocal mutated gene, the study of genetic variants appears to better meet researcher expectations even when such variants actually cannot be paired with the unilateral manifestation of ISSHL. In addition, previous studies have reported the correlation of various polymorphisms with an increased [39–43] or reduced [44] risk of developing hearing impairment. Thus, currently, the definition of new pathways of molecular interactions is essential in supporting genetic observations (Figures 1–3).

Recent advanced findings in regards to ROS-mediated damage and nitric oxide (NO) activity have encouraged researchers to focus their studies on possible connections between microvascular damage and free radical production. The study of polymorphisms of genes related to iron metabolism in relation to SSNHL has not been previously reported: we hypothesized that these variants could also be involved in the pathogenesis of SSNHL [45]. Nonetheless, the role of ferroportin in managing the delicate iron redox balance at the level of the cochlea or the auditory nerve is still unclear [46]. However, it is possible to assume that, under stressful conditions, cells need to rapidly reduce the intracellular free divalent iron pool, and this can produce oxidative damage. Moreover, increased levels of Fe^2+^ inhibit the intracellular production of NO, a fundamental element in the regulation of local microcirculation. Nitric oxide has a decisive role in the regulation of cochlear flow. We can divide the inner ear into four large anatomofunctional districts: (1) homeostasis of labyrinthine fluid, (2) stria vascularis, (3) organ of Corti, and (4) neurons and spiral ganglion. Mitochondria could be considered as the center of connection of these parts or as a fifth district [47–49]. Thus, unilateral sudden hearing loss might be considered the consequence of recurrent aggression of important inner ear structures that, in the end, leads to local damage with trigger points that are linked to general clinical conditions, comorbidity, and functioning local anatomy. These elements might explain the age of onset and unilateral expression of the disease.

The auditory system might be optimized to minimize the persistence and the concentration of divalent metal ions in the inner ear fluids [50]. This homeostatic system is finely tuned to prevent, ultimately, damage to the sensorineural cells, without compromising the function of vital processes dependent on the presence of iron, especially at the mitochondrial level (Figure 3). Studying the location of other proteins involved in the metabolism of iron (ferritin, transferrin, and* DMT1*), we determined a precise localization of the ferroportin in the associated pathway and speculated on possible effects of the polymorphism* FPN1* −8CG in the promoter area (Figure 2). This polymorphism belongs to a group of iron gene variants previously investigated by our group in other complex diseases in which iron-driven inflammation or anomalous redox balance may have a role or be suspected (10, 13, 14, 19). On the basis of the assumption that they are not the only ones responsible for the disease, they could instead act together with something not completely known (often acquired but not exclusively) and account for the establishment of the pathological condition. In detail, the five variants are relatively common in the racial group investigated and are selected just to sound our theory not considering this fact as definitive and completely exhaustive. Finally, having performed in this study a case-control comparison in which the two groups completely matched for sex, age, and ethnicity, we can exclude that the overrepresentation of the allele* FPN1-8G,* observed among cases, could be due to a particular ethnicity who selects a particular gene variant or to other influencing factors.

Accordingly, NO, free radicals (ROS), and NF*κ*B might potentially be involved during inner ear fluid homeostasis. The interaction of these elements ultimately involves other proteins with the precise purpose of regulating the local inflammatory response, blood flow, and cellular activity (including mitochondrial activity) [51–54]. For example, the stria vascularis, in its basal layer and the intermediate layer, reacts mainly toward a response to inflammation mediated by NF*κ*B [55–57]. In another district or below a certain degree of local aggression, an antioxidant response mediated by NFRE-2 is favored, especially at the level of the organ of Corti.

The expression of ferritin [58] and the concomitant apical expression of* DMT1* [59] suggest that the stria vascularis has an active role in restoration and deposition of iron, with particular attention to reducing the endolymphatic concentration of divalent ions [60, 61]. Accordingly, it is known that free radical stress-mediated loss of specific protein expression (i.e.,* Kcnj10*) in stria vascularis contributes to hearing loss in Pendred syndrome mouse model [18]. Pendred syndrome, the most frequent hereditary form of syndromic deafness, is caused by loss-of-function mutations of the gene* SLC26A4*, which encodes the anion exchanger pendrin [62]. Transient/constitutive reduction of* Kcnj10 *protein expression under free radical stress could be the pathobiologic mechanism of sudden hearing loss that could be not limited just to Pendred syndrome.* SLC26A4 *and* SLC40A1* genes belong to the same (super)family and might be involved, by regulating inflow/outflow of different ions, to maintain specific homeostasis and redox potency [63]. Stria vascularis is sensitive to free radical stress due to its high metabolic activity and dense vascular system [64] and when iron-chelator proteins are not adequately functioning or are not enough to contrast and neutralize the detrimental effect of iron this situation could dramatically affect the optimal stria vascularis functions.

We could also hypothesize/speculate a mechanism of supplementation of divalent ions into the endolymphatic space affecting the volume and the acidification of the endolymph, both affected in the Pendred syndrome. Nevertheless, this role is most likely less important than the reuptake because of the endolymphatic electrical gradient.

Iron is predominantly bound to transferrin that is present in increasing respective concentrations in perilymph, cerebrospinal fluid, and blood [65–67]. This could explain the intracytoplasmic expression and localization of* DMT1* in the organ of Corti. Conversely,* DMT1* is absent at the level of the ciliated cells and highly expressed at the level of neuronal fibers and in particular of the basal ganglion. Summarizing, changes in the redox capacity of a system affect indeed the pH with drastic biological effects. These data suggest a similar involvement of ferroportin as indicated above and are in agreement with kidney analogous distribution [68]. Furthermore, the receptors for transferrin might mediate the internalization of vesicles in ciliated cells by TfR [69]. These receptors must be absent in the ciliated cells, and the iron may enter in a minimum amount, finely controlled, most likely through the basolaterally expressed calcium channels in ciliated cells: L-type calcium channels, localized basolaterally in hair cells that could allow the intake of a few molecules of Fe^2+^ [70–76]. An intercellular passage at the level of the inner ear through the gap junctions cannot be excluded. In fact, the diameter of the channels formed by the connection would allow the passage of molecules such as divalent metal ions, as these, especially in the case of the iron, are less than 2 nm in diameter and very close in size to calcium ions that habitually take advantage of the gap junction for cytoplasmic passage from cell to cell [77]. The gradient that the free iron could exploit is that of bidirectional facilitated diffusion: iron might be able to “spreading out” in case of accumulation and “coming back” in case of consumption if necessary. According to this circle, this type of iron distribution calls to mind in some aspects the recirculation of the potassium ions but most likely with opposite direction. The longitudinal gradient that is already physiologically present along the cochlea (the concentration of endolymphatic potassium increases from the apex to the base) might play a role in the flow of divalent ions [78], especially in pathological conditions.

Summarizing, FPN1 −8CG gene polymorphism displayed a significant association with ISSNHL, as patients carrying this polymorphism displayed an increased risk of developing this disease in adulthood. The distribution of proteins involved in the recirculation of divalent metal ions leads us also to believe that ferroportin is coupled to the expression of* DMT1*. The distribution of* DMT1* is superficial at the level of the stria vascularis where we find the largest concentration of ferritin [58, 79]. The task of reabsorption of divalent metals, including iron, with the possibility of storage, is most likely performed by the stria vascularis [80]. On the contrary, the nerve fibers, spiral ganglia of the organ of Corti (with the exception of ciliated cells internal and external), display cytoplasmic localization of* DMT1 *[81]. This suggests a system of mobilization of iron, or of other divalent metals, mediated by transferrin, which, in fact, is more represented in the perilymph and in the cerebrospinal fluid, which is in contact with the organ of Corti and nerve bundles.

Conversely, the outer and inner hair cells do not appear to express* DMT1*. This does not necessarily exclude the expression of* FPN1* at this level. Nevertheless, it suggests how neurosensory cells might be excluded as much as possible from redox activities, inflammatory responses, and ionic movements, most likely because of their specific nature.

Ferroportin is the main mammalian iron exporter, and by means of the homozygous gene variant condition (−8GG) could affect the local iron levels poisoning microenvironment. In this context, it is reasonable to assume that the polymorphism, by affecting the expression of ferroportin, could modify the iron homeostasis at the local level. Though the nucleotide substitution is not strictly close to the IRE (iron regulatory element) region of the gene, it is in strong disequilibrium with the −98GC variant (Figure 2) that is instead more close [82]. Then, by virtue of the documented high conservation of this promoter gene area among mammals (Figure 1), it could be possible that one or both of these variants dysregulated optimal FPN1 function by affecting the IRE-IRP signaling pathway. In addition, it is to take into account that macrophages carry on their surface the FPN1 receptor and that they contribute to the degeneration of the stria vascularis in Pendred syndrome [83]. Accordingly, an altered macrophage action could further worsen the critical homeostasis in terms of iron overload and related oxidative-stress as previously suggested in different degenerative diseases considering that the “*mutated*” macrophages might increase the possibility of generating free iron and free radicals, possibly leading to matrix breakdown and cell death [14, 84] (Figure 3).

In conclusion, further larger epidemiological studies are required to elucidate the pathophysiological mechanisms. Such a complex disease is to consider a multifactorial and polygenic condition in which gene-environment interactions have a key role. In particular, on the basis of the iron-driven hypothesis and that of the oxidative stress, additional correlated gene variants could be considered as well as those involved in the homocysteine, metalloprotease, or hemopexin pathways possibly playing a role in this complex biochemical mechanism.

## Figures and Tables

**Table 1:** Main characteristics of the population investigated.

	Cases	Controls	*P* value
Number	**200**	**400**	—
Age mean ± SD median, range	63.65, (10–92)	63.5, (10–92)	NS
Male/female	96/104	190/210	NS
Left side	95	—	—
Right side	103	—	—
Bilateral	2	—	—
Mild hearing loss	44	—	—
Moderate	92	—	—
Severe	33	—	—
Profound	31	—	—

**Table 2:** Gene variants distribution and associated ORs values. The ORs have been calculated comparing the number of the polymorphic homozygotes with the rest of genotypes among cases versus the controls.

Gene variant	*FPN1 (SLC40A1)*			*Tf*			*HFE*			*HFE*		*HEPC (HAMP) *		
	*−8CG *			*C1, C2 *			*H63D *			*C282Y *		*−582AG *		

Cases	200			200			200			200		200		
Controls	400			400			400			400		400		

Genotypes	CC	CG	GG	C1C1	C1C2	C2C2	HH	HD	DD	CC	CY	AA	AG	GG

Cases	123	62	15	139	53	8	142	56	2	193	7	111	77	12
%	*61.5 *	*31 *	*7.5 *	*69.5 *	*26.5 *	*4 *	*71 *	*28 *	*1 *	*96.5 *	*3.5 *	*55.5 *	*38.5 *	*6 *
Controls	268	125	7	270	119	11	294	98	8	383	17	229	147	24
%	*67 *	*31.25 *	*1.75 *	*67.5 *	*29.75 *	*2.75 *	*73.5 *	*24.5 *	*2 *	*95.75 *	*4.25 *	*57.25 *	*36.75 *	*6 *
*P* value	***0.001***			*0.63 *			*0.55 *			*0.80 *		*0.92 *		
OR (95% CI)	**4.27 (2.65–6.89)**			1.39 (0.71–2.73)			0.52 (0.13–2.08)			0.82 (0.33–2.0)		1.03 (0.60–1.76)		

Alleles	C	G		C1	C2		H	D		C	Y	A	G	

Cases number/%	308/77	92/23		331/82.75	69/17.25		340/85	60/15		393/98.25	7/1.75	299/74.75	101/25.25	
Controls number/%	661/82.6	139/17.4		659/82.4	141/17.6		686/85.7	114/14.3		783/97.9	17/2.1	605/75.6	195/24.4	
*P* value	***0.02***			*0.94 *			*0.79 *			*0.83 *		*0.79 *		
OR (95% CI)	**1.42 (1.06–1.91)**			0.97 (0.71–1.34)			1.06 (0.76–1.49)			0.82 (0.34–1.99)		1.05 (0.8–1.38)		

**Table 3:** Hearing loss severity score stratified by different genotypes among the different gene variants: 44 (mild), 92 (moderate), 33 (severe), and 31 (profound). HL severity score is graded in the following: mild hearing loss (16–40 dB HL), moderate hearing loss (41–70 dB HL), severe hearing loss (71–90 dB HL), and profound hearing loss (>90).

Gene variant	*FPN1 (SLC40A1) *			*Tf *			*HFE *			*HFE *		*HEPC (HAMP) *		
	*−8CG *			*C1, C2 *			*H63D *			*C282Y *		*−582AG *		

Cases	200			200			200			200		200		

Genotypes	CC	CG	GG	C1C1	C1C2	C2C2	HH	HD	DD	CC	CY	AA	AG	GG

Total (*n*)	123	62	15	139	53	8	142	56	2	193	7	111	77	12

HL severity score (PTA dB HL)														
16–40 dB HL	27	12	5	27	16	1	31	13	0	42	2	34	6	4
41–70 dB HL	60	26	6	66	24	2	62	28	2	88	4	51	35	6
71–90 dB HL	18	12	3	21	8	4	25	8	0	32	1	12	19	2
>90 dB HL	18	12	1	25	5	1	24	7	0	31	0	14	17	0
